# Investigation on quality of life of parents of children with cerebral palsy and its influencing factors: A cross-sectional study

**DOI:** 10.1097/MD.0000000000045441

**Published:** 2025-10-31

**Authors:** Ling Wan, Lin Jiang, Sha Chen, Minghui Yin, Qiong He

**Affiliations:** a Wuhan Children’s Hospital (Wuhan Maternal and Child Healthcare Hospital), Tongji Medical College, Huazhong University of Science & Technology, Wuhan, Hubei, China.

**Keywords:** cerebral palsy, children, coping styles, parents, psychological stress, quality of life, social support

## Abstract

This study investigates the level of quality of life among parents of children with cerebral palsy (CP) and provides a reference for improving intervention measures to enhance their quality of life. A convenience sample of 160 parents of children with CP were selected, and a questionnaire survey was conducted using The World Health Organization Quality of Life-Based Rating Scale, the psychological stress questionnaire for parents of children with disabilities (OMS-PDC), the Simple Coping Styles Questionnaire and the Perceived Social Support Scale. The quality of life score for parents of children with CP was (61.53 ± 11.73) points. The quality of life of parents was positively correlated with coping style and social support (all *P* < .001). The relationship with the child, average monthly family income, cumulative rehabilitation time, psychological stress, coping style, and social support were important factors affecting the quality of life of parents of children with CP, explaining 37.70% of the variability in quality of life. The quality of life of parents of children with CP is at a moderate level and is influenced by various factors such as the relationship with the child, average monthly family income, cumulative rehabilitation time, psychological stress, coping style, and social support. In clinical work, attention should be paid to the quality of life and physical and mental health of parents of children with CP in order to promote their rehabilitation.

## 1. Introduction

Cerebral palsy (CP) is caused by congenital developmental defects (malformations, intrauterine infections) or acquired (prematurity, low birth weight, asphyxia, hypoxic-ischemic encephalopathy, kernicterus, trauma, infections) and other nonprogressive brain damage in the immature brain (prenatal, natal, or postpartum) and is manifested mainly by motor deficits, with or without perceptual and intellectual. The main manifestations are motor deficits, with or without perceptual and intellectual deficits.^[1,2]^ CP is a common motor disorder in children, which has the characteristics of long course, slow recovery and high disability rate. With the progress of neonatal intensive care technology, the survival rate of high-risk infants increased significantly, but the incidence of CP in children also showed an upward trend. There is no effective cure for CP, and functional rehabilitation is currently recognized.^[3]^ Children with CP not only need long-term treatment and rehabilitation training but also need parents’ long-term company and care. The long-term rehabilitation treatment has brought heavy economic burden and huge psychological pressure to the parents of children with CP, which has attracted extensive attention worldwide. As the caregiver and rehabilitation treatment assistant of children with CP, the quality of life and physical and mental health status of the parents of children with CP are closely related to the rehabilitation process of the children. Therefore, it is of great significance to deeply understand and improve the quality of life of the parents of children with CP to promote the rehabilitation of children with CP. In recent years, the research on the quality of life of parents of children with CP has gradually increased. A number of studies at home and abroad have comprehensively evaluated the quality of life of parents of children with CP from multiple dimensions such as physiology, psychology and society, and revealed that the quality of life of parents of children with CP is generally lower than that of parents of healthy children. Other studies have explored many factors that affect the quality of life, such as economic pressure, insufficient social support, mental health problems, cultural differences and social environment, but the deep-seated reasons still need to be explored. This study investigated the quality of life of parents of children with CP, analyzed the correlation between quality of life and psychological stress, coping style, social support and other factors, and further explored the influencing factors of quality of life, so as to provide theoretical basis for targeted improvement of parents’ quality of life in the later stage, so as to bring more hope and warmth to the families of children with CP, and provide more powerful support for the rehabilitation of children with CP.

## 2. Methods

### 2.1. Study design

This study employed the cross-sectional study. This study was performed after receiving approval from the institutional review board of Wuhan Children’s Hospital (2020R031-E01). All information of the research participants is ensured to be kept confidential. The respondents had full right to refuse to take part in the study, and this was clearly explained as part of the information sheet.

### 2.2. Setting

The data collection time of this study was from January 2022 to October 2022. The children with CP and their parents in the Department of rehabilitation medicine of our hospital were selected as the research objects.

### 2.3. Participants

Inclusion criteria: meet the diagnostic criteria of pediatric CP^[4]^; the age of the children was ≤12 years old; the parents of the children were the main caregivers; the children received standardized rehabilitation treatment in our hospital and complied with the doctor’s instructions well; the parents of the children had elementary school education or above, and they were able to fill in the questionnaires on their own. Exclusion criteria: the parents of the children had cognitive, hearing or speech disorders or a history of mental illness. Informed consent was signed by all study participants. A total of 160 children and their parents’ data were collected in this study. Ninety-two children were male and 68 were female; the age of the children ranged from 10 to 65 months, and the average age of the children was (38.29 ± 10.74) years old. Type of CP: 112 cases of spastic type, 32 cases of involuntary movement type, 12 cases of mixed type and 4 cases of ataxia type. Duration of the disease ranged from 5 to 40 months, with an average duration of (21.63 ± 7.14) months. The cumulative time of training in professional rehabilitation institutions ranged from 1 to 33 months, with an average of (17.36 ± 6.53) years; among them, 142 cases had been adhering to rehabilitation (>90% of the number of times of the rehabilitation program was achieved), and there were 18 cases with interruptions or not adhering to rehabilitation (>10% of the number of interruptions of the rehabilitation program). There were 132 mothers and 28 fathers of the affected children; their ages ranged from 22 to 45 years old; the average age was (30.91 ± 4.55) years old. Educational level: 63 in junior high school and below, 46 in high school/secondary school, 51 in college/undergraduate school and above. Per capita monthly household income: <3000 yuan for 45 people, 3000 to 5000 yuan for 82 people, >5000 yuan for 33 people.

### 2.4. Data sources

#### 2.4.1. General information questionnaire

Self-designed questionnaire, including 2 parts: data of children: age (divided into <24 months and ≥24 months), gender (divided into male and female), type of CP (divided into spastic type, involuntary movement type, mixed type, ataxia type), course of disease (divided into <12 months and ≥12 months), cumulative time of rehabilitation treatment (divided into <12 months and ≥12 months), rehabilitation treatment (divided into continuous rehabilitation, interrupted or not). Parents’ information: relationship with children (divided into mother and father according to the gender of parents), parents’ age (divided into <30 years old and ≥30 years old with the age of 30 as the dividing point), education level (divided into junior high school and below, high school/technical secondary school, college/undergraduate and above), family per capita monthly income (divided into <3000 yuan, 3000–5000 yuan and >5000 yuan), monthly rehabilitation expenses (divided into <5000 yuan and ≥5000 yuan with the age of 500 yuan as the dividing point), parents’ CP knowledge level (divided into low level, medium level, and high level).^[5–7]^

#### 2.4.2. The World Health Organization Quality of Life-Based Rating Scale

The World Health Organization Quality of Life-Based Rating Scale consists of 26 items in 4 dimensions: physiological (7 items), psychological (6 items), social relations (3 items), and environmental (8 items), as well as 2 independently analyzed subjective items, that is, general health status and quality of life.^[8]^ Each entry was scored from 1 to 5, including “very poor/very dissatisfied,” “poor/dissatisfied,” “fair,” “good/satisfied,” and “very good/satisfied.” “Fair,” “Good/Satisfactory,” and “Very Good/Highly Satisfactory.” Each dimension was assessed on a percentage scale, with the higher the score the better the quality of life. The Cronbach alpha coefficient for the total scale was 0.88.

#### 2.4.3. OMS-PDC

OMS-PDC consists of 33 items in 5 dimensions: personal and family problems (9 items), financial burden (7 items), lifelong care (6 items), lack of fulfillment (7 items), and overprotection (4 items). A self-assessment format was used with 2 choices of “yes” and “no” for each question. The choice of One of these choices was scored as 0.1. The high and low cumulative scores indicate the level of parental psychological stress, respectively.^[9]^ The Cronbach alpha coefficient for the total scale was 0.80.

#### 2.4.4. Simple Coping Styles Questionnaire

Simple Coping Styles Questionnaire containing 20 items in 2 dimensions: positive coping styles (12 items), negative coping styles (8 items), each item counts from 0 to 3 points (never, occasional, sometimes, and often), and the total score ranges from 0 to 60, with higher scores indicating a greater tendency toward that coping style.^[10]^ The Cronbach alpha coefficient for the total scale was 0.79.

#### 2.4.5. Perceived Social Support Scale

Perceived Social Support Scale developed by Zimet et al and introduced and revised by Jiang Qianjin et al.^[11,12]^ It is a social support scale that emphasizes the individual’s self-understanding and self-feelings, and contains 12 entries in 3 dimensions, family support (4 entries), friend support (4 entries), and other support (4 entries), each of which is scored from 1 to 7 (strongly disagree, strongly disagree, slightly disagree, neutral, slightly agree, strongly agree, and strongly agree). The Cronbach alpha coefficient for the total scale was 0.87.

### 2.5. Study size

The questionnaire collection was carried out by 4 investigators, who were uniformly trained before the start of the project and skilled in the methods and precautions of questionnaire collection. The survey explained the significance of the purpose of the survey and the method of filling out the questionnaire. The questionnaires were filled out independently by the research subjects, answered anonymously, and the questionnaires were collected on site. In this study, a total of 167 cases were investigated and 160 valid questionnaires were recovered, with an effective rate of 95.81%. One primary caregiver (father or mother) was investigated in each case.

### 2.6. Statistical methods

SPSS 22.0 statistical analysis software (IBM Corp., Armonk) was used. The quality of life score, psychological stress score, coping style score and social support score were the measurement data. The Shapiro–Wilk normality test showed that they conformed to the normal distribution (*P* > .05), expressed as the mean ± standard deviation (*x* ± *s*). The independent sample *t* test was used for the comparison between the 2 groups, and the analysis of variance was used for the comparison between multiple groups. According to the purpose of this study, the quality of life score, psychological stress score, coping style score and social support score were included in Pearson correlation analysis to analyze the correlation between the above factors. The relationship with children, parents’ education level, family per capita monthly income, monthly rehabilitation costs, rehabilitation treatment, cumulative time of rehabilitation, psychological stress, coping style, and social support were taken as independent variables, and the total score of quality of life was taken as dependent variable. The above multivariate analysis was performed by multiple linear regression analysis. The difference was statistically significant (*P* < .05).

## 3. Results

### 3.1. Quality of life and psychological stress, coping styles, and social support scores of the parents of the affected children

The total quality of life scores of children with CP ranged from 31 to 96 (61.53 ± 11.73), and the quality of life of their parents was at the medium level. The total scores and dimension scores of each scale of psychological stress, coping styles, and social support are shown in Table 1.

**Table 1:** Quality of life and scores of psychological stress, coping styles, and social support of the parents of the children ($\bar{x}\pm \;s$, points).

Item	Total points ($\bar{x}\pm \;s$)	Average score ($\bar{x}\pm \;s$)
Quality of life	61.53 ± 11.73	2.37 ± 0.45
Physiological field	14.24 ± 3.12	2.38 ± 0.52
Psychological field	13.60 ± 3.29	2.27 ± 0.55
Social relations field	13.87 ± 3.08	2.31 ± 0.52
Environmental field	14.98 ± 2.98	2.50 ± 0.50
Overall health status and quality of life	4.85 ± 1.29	2.42 ± 0.65
Mental stress	13.27 ± 3.3	0.40 ± 0.10
Coping style	31.39 ± 7.99	1.57 ± 0.40
Social support	60.07 ± 12.91	5.01 ± 1.08

### 3.2. Univariate analysis of quality of life of parents of children with

The difference in the quality of life scores of parents of children with CP grouped by different factors was statistically significant (*P* < .05) in terms of relationship with the child, parents’ literacy, per capita monthly family income, monthly rehabilitation costs, rehabilitation treatment, and cumulative duration of rehabilitation. In terms of parents’ age, parents’ knowledge of CP, children’s age in months, duration of children’s illness, and type of children’s CP, the differences in the quality of life scores of parents of children with CP grouped by different factors were not statistically significant (*P* > .05), as shown in Table 2.

**Table 2:** Univariate analysis of the quality of life of the parents of the affected children (n = 160).

Factors	n	WHOQOL-BREF score	*t*/*F*	*P*
Relationship with children			2.892	.004
Mother	132	60.32 ± 11.38		
Father	28	67.22 ± 11.88		
Parental age (yr)			1.124	.263
<30	67	62.73 ± 11.72		
≥30	93	60.62 ± 11.72		
Parental education			10.490	<.001
Junior high school and below	63	58.79 ± 11.65		
Senior high school/technical secondary school	46	58.10 ± 9.79		
Junior college/bachelor degree or above	51	67.12 ± 11.48		
Average monthly family income (yuan)			13.351	<.001
<3000	45	56.55 ± 10.97		
3000~5000	82	66.92 ± 12.04		
>5000	33	66.41 ± 9.48		
Monthly rehabilitation cost(yuan)			2.112	.036
<5000	83	63.42 ± 12.51		
≥5000	77	59.49 ± 10.89		
Parents cerebral palsy knowledge level			1.074	.344
Low level	59	59.88 ± 9.65		
Middle level	82	62.19 ± 13.11		
High level	19	63.81 ± 11.19		
Month age of child (mon)			0.176	.860
<24	52	61.77 ± 13.17		
≥24	108	61.42 ± 11.04		
Disease course of child (month)			1.006	.316
<12	55	62.82 ± 12.98		
≥12	105	60.85 ± 11.08		
Types of cerebral palsy in children			2.470	.064
Spastic	112	62.80 ± 11.93		
Dyskinetic	32	58.01 ± 11.18		
Mixed	12	56.98 ± 9.43		
Ataxia	4	67.88 ± 9.00		
Rehabilitation treatment			2.696	.008
Always insist	118	60.02 ± 12.77		
Discontinuity/no adhere to	42	65.77 ± 8.82		
Cumulative rehabilitation time (mon)			2.913	.004
<12	74	64.03 ± 11.84		
≥12	86	58.62 ± 11.60		

WHOQOL-BREF = The World Health Organization Quality of Life-Based Rating Scale.

### 3.3. Correlation of quality of life of parents of children with psychological stress, coping styles, and social support

**Figure 1.**
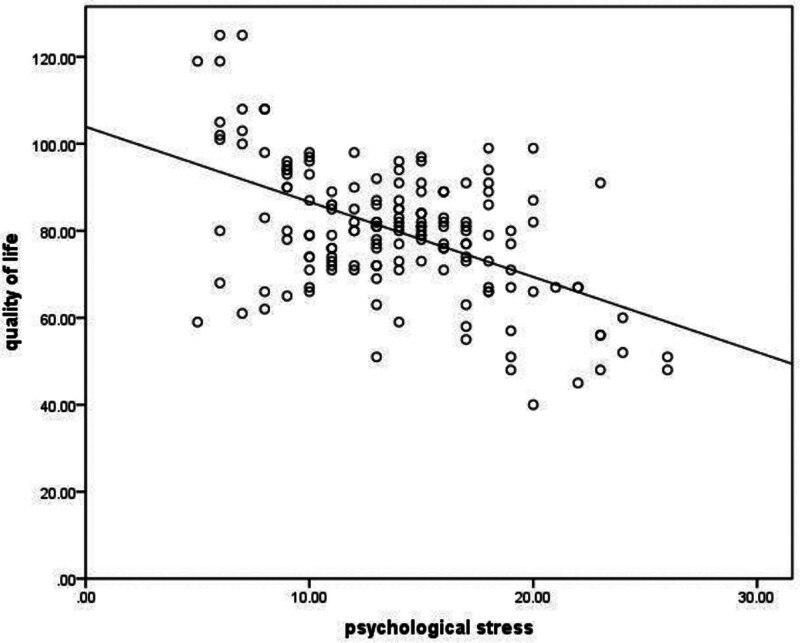
The quality of life is negative correlated with psychological stress.

**Figure 2.**
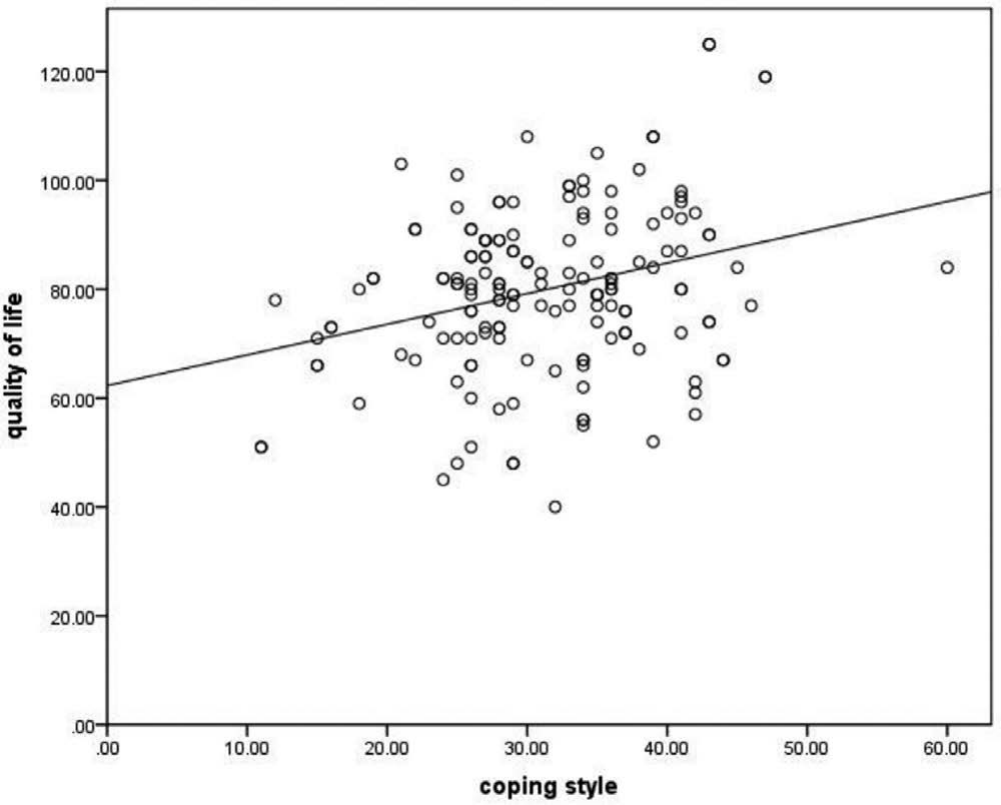
The quality of life is positively correlated with coping style.

**Figure 3.**
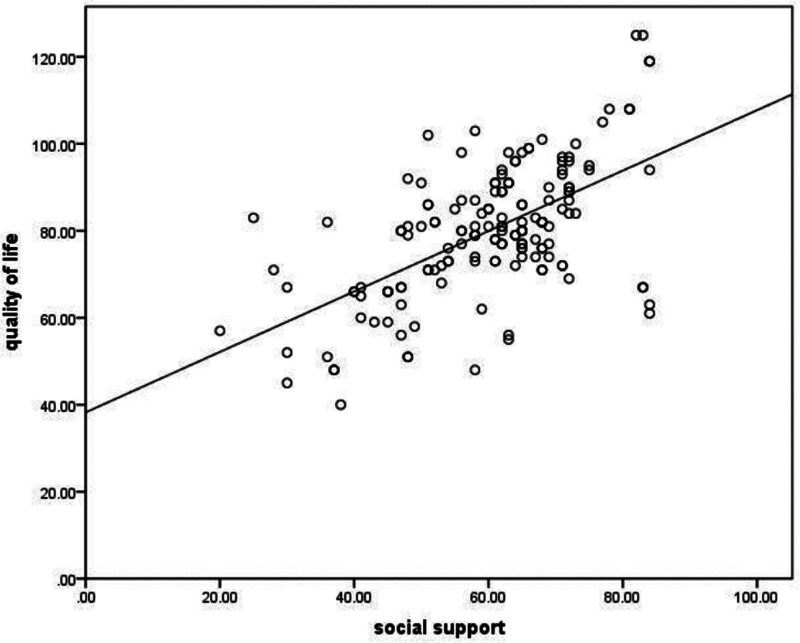
The quality of life is positively correlated with social support.

Pearson correlation analysis showed that the quality of life of the child’s parents was negatively correlated with psychological stress (*r* = −0.420, *P* < .001); the quality of life of the child’s parents was positively correlated with coping styles and social support (*r* = 0.295, *r* = 0.589, both *P* < .001), as shown in Figures 1–3.

### 3.4. Regression analysis of factors influencing the quality of life of the parents of the affected children

**Figure 4.**
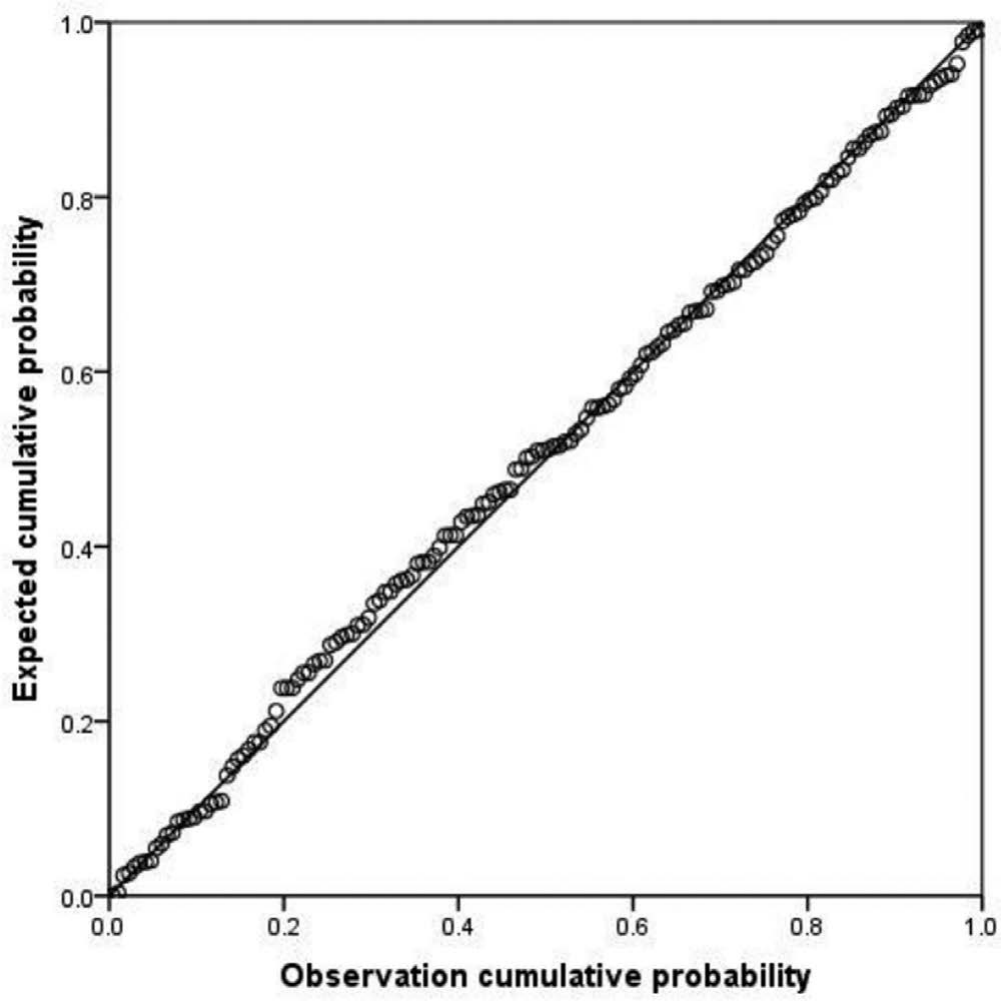
Observation and expected cumulative probability.

**Figure 5.**
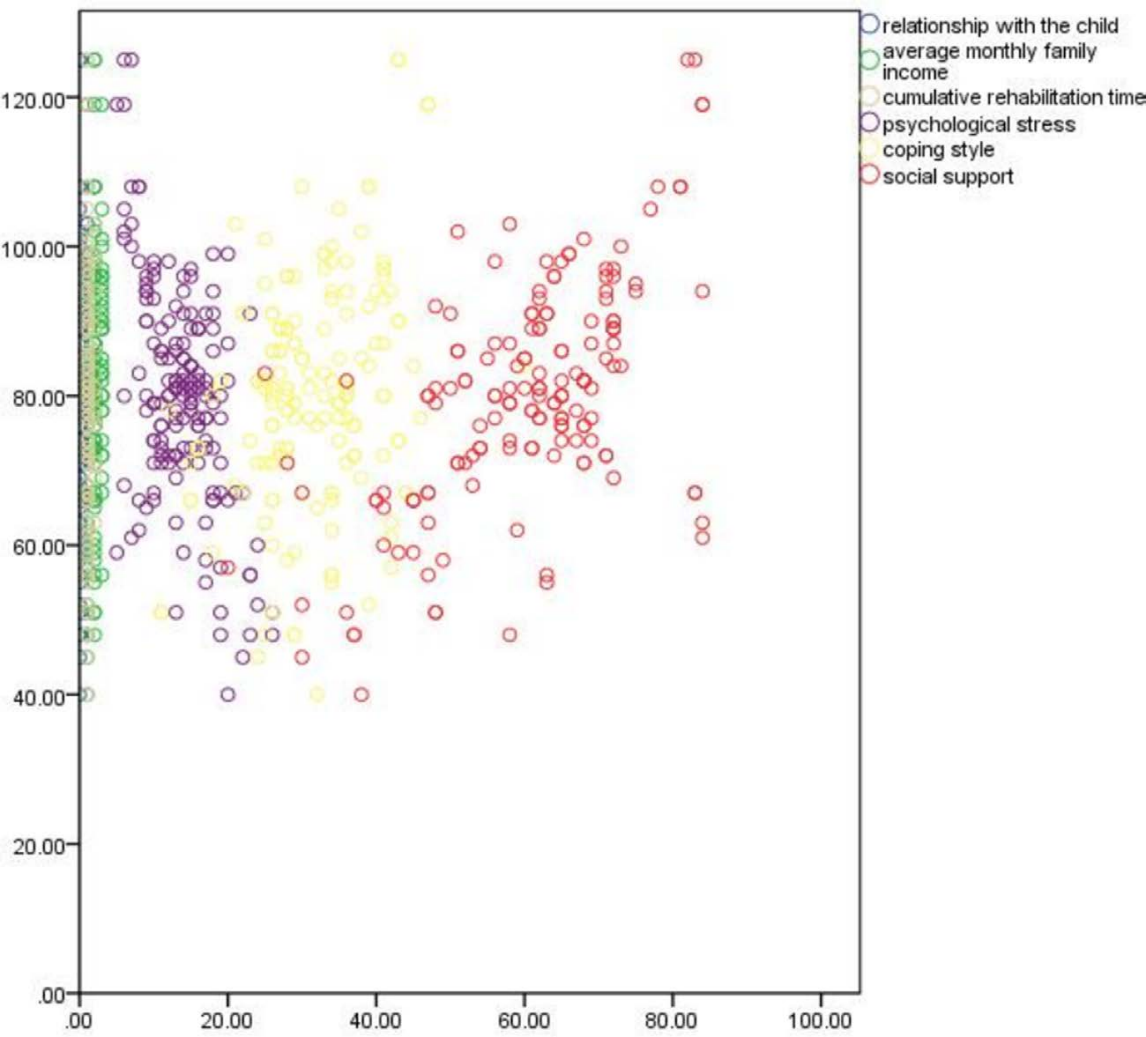
Regression chart of the relationship between 6 influencing factors and quality of life.

Multiple linear regression analyses were performed using the total quality of life as the dependent variable and the factors that were statistically significant in the univariate analysis as the independent variables, according to the criteria of alpha in = 0.05 and alpha out = 0.10. The values assigned to the independent variables are shown in Table 3. The results showed that relationship with the child, per capita monthly income of the family, cumulative time of rehabilitation, psychological stress, coping styles, and social support were significant factors affecting the quality of life of parents of children with CP, which explained 37.70% of the variance in the quality of life. The results of regression analysis are shown in Table 4 and Figures 4 and 5.

**Table 3:** Assignment of independent variables.

Variables	Assignment
The relationship with the child	Mother = 0, father = 1
Parental education	Junior high school and below = 0 Senior high school/technical secondary school = 1, junior college/bachelor degree or above = 0 Senior high school/technical secondary school = 0, junior college/bachelor degree or above = 1
Average monthly family income	<3000 = 0 3000–5000 = 0, ≥5000 = 1 3000–5000 = 1, ≥5000 = 0
Monthly rehabilitation cost	≥5000 = 0, <5000 = 1
Rehabilitation treatment	Always insist = 0, discontinuity/no adhere to = 1
Cumulative rehabilitation time	≥12 months = 0, *<*12 months = 1
Mental stress	Actual value
Coping style	Actual value
Social support	Actual value

**Table 4:** Results of regression analysis.

Variables	*β*	*SE*	*β’*	*t*	*P*
Constant	21.713	4.695	–	4.625	<.001
The relationship with the child	6.694	2.566	0.167	2.608	.010
Parental education	0.523	1.056	0.037	0.495	.621
Average monthly family income	2.864	1.075	2.098	4.187	.008
Monthly rehabilitation cost	2.039	1.614	0.093	1.263	.208
Rehabilitation treatment	0.824	0.735	0.280	1.121	.263
Cumulative rehabilitation time	1.997	0.852	1.367	2.343	.020
Mental stress	−0.271	0.045	−0.119	6.022	<.001
Coping style	0.413	0.133	0.051	3.105	.002
Social support	0.599	0.085	0.508	7.032	<.001

*R*^2^ = 0.400, adjusted *R*^2^ = 0.377; *F* = 17.026, *P* < .001.

## 4. Discussion

### 4.1. Level of quality of life of parents of children with CP

The results of this study showed that the quality of life score of parents of children with CP was (61.53 ± 11.73), which was in the middle level. Because of the long time and high cost of rehabilitation treatment for children with CP, a sound rehabilitation treatment and rescue system should be established to reduce the burden on families, improve the quality of life of parents of children with CP, and ensure that the children receive effective rehabilitation.^[13]^ The quality of life of the caregivers, in turn, is directly related to the quality and effectiveness of the child’s rehabilitation, thus affecting the quality of life of children with CP.^[14]^ Therefore, it is necessary to understand the quality of life of parents of children with CP and their influencing factors, and to improve the unfavorable factors affecting the quality of life of parents of children with CP through social, organizational, and policy interventions to improve the quality of their lives and enhance the ability to support their families, so as to achieve the goal of improving the accessibility and effectiveness of rehabilitation services for children with CP.^[15]^

### 4.2. Factors affecting the quality of life of parents of children with CP

#### 4.2.1. Relationship with the affected child

In this study, 17.5% were fathers and 82.5% were mothers, and most of those responsible for the rehabilitation of their children were mothers. The results of this study showed that the quality of life of mothers was poorer than that of fathers, which is consistent with the study reported by Yilmaz.^[16]^ It may be due to the fact that: children with CP have a long period of treatment and rehabilitation, which requires great patience and love from their caregivers, and women are more attentive and patient compared to men; this means that mothers are in a more fragile psychological situation among parents of children with CP, which is the main reason for their quality of life, and they should be given proper attention and effective measures to improve it.

#### 4.2.2. Monthly per capita household income

The results of this study showed that per capita monthly family income had a significant effect on the quality of life of the parents of the affected children, and the lower the income, the worse the quality of life. Due to the poorer economic situation, it is difficult to effectively guarantee the rehabilitation treatment of the children, which reduces the sense of control over the children’s diseases and leads to the low level of quality of life of the parents of the children. On the other hand, parents of children with higher per capita monthly family income are in a relatively favorable economic situation and can provide good conditions for their children to receive medical treatment, which can effectively guarantee the long-term rehabilitation treatment of their children and therefore have a higher quality of life.

#### 4.2.3. Rehabilitation cumulative time

The results of this study showed that the longer the cumulative time of rehabilitation, the worse the quality of life. CP belongs to the children’s chronic disease, the course of the disease is long, need to carry out long-term comprehensive rehabilitation treatment, the high cost of treatment will bring a heavy financial burden to the parents of the children. Not only do they have to bear the double pressure of the child’s disease and rehabilitation treatment, but they also have to bear the heavy financial burden brought by the child’s disease. Therefore, the longer the cumulative time of rehabilitation, the worse the quality of life of the parents.

#### 4.2.4. Psychological stress

The results of this study show that psychological stress is also an important factor affecting the quality of life of parents of children with CP. Carers of children with disabilities generally have excessive burden of care, economy, etc^[17]^ and face great pressure of parenting and caring, long-term rehabilitation treatment but it is difficult to see obvious results, parents bear pressure from the child, within the family and the society, etc, which can easily lead to physical and mental exhaustion, affecting their quality of life. Medical personnel should provide appropriate psychological assistance to parents to relieve their psychological pressure.

#### 4.2.5. Response modalities

The average age of the children in this study was (38.29 ± 10.74) months, which is relatively young, and they received treatment earlier. Parents may think that they have a higher vision of their children’s treatment, rehabilitation and future, and they can view the disease optimistically, actively cooperate with medical personnel, and take the initiative to obtain, analyze and process information related to their children’s disease, which will help parents to construct a cognitive framework of their children’s disease. This will help parents to construct a cognitive framework for their children’s illnesses and improve the quality of life of parents of children with CP to a certain extent.

#### 4.2.6. Social support

The results of this study show that a good social support system can improve the quality-of-life level of parents of children with CP and promote better cooperation between parents of children with CP and rehabilitation treatment and care. Social and family support in terms of spiritual and economic help is especially needed. Therefore, healthcare professionals should provide psychological counseling to the parents of children with CP, and carry out effective communication and exchanges, so as to encourage the parents of children with CP to be emotionally stable and actively cooperate with the treatment and promote the recovery of their children.

## 5. Conclusion

The quality of life of parents of children with CP is at a moderate level and is influenced by a variety of factors such as relationship with the child, per capita monthly family income, cumulative time in rehabilitation, psychological stress, coping styles, and social support. The physical and mental health of parents of children with CP is an important guarantee for the follow-up treatment of children with CP, so identifying the factors affecting parental healthiness and well-being is essential for the child’s recovery.^[18]^ At the same time, strengthening communication with parents of children with CP, relieving their psychological pressure, instructing them to face the disease with a positive attitude, and giving certain social support and help. This study also has some limitations. On the one hand, due to the limitation of clinical conditions, the sample size of cases collected in this study is small, which may have sampling bias, leading to the error of statistical results, and the influencing factors screened out are also less, which is difficult to accurately reflect the influencing factors of the quality of life of parents of children with CP; on the other hand, the regional sources of cases in the single center sample are single, and the representativeness is obviously limited, which is difficult to be extended to other regions. It is suggested that in the future, multi center and large sample studies should be carried out to analyze more influencing factors, and a prediction model for the quality of life of parents of children with CP should be constructed to further guide medical staff to help parents of children with CP improve the quality of life, so that parents can face the rehabilitation treatment of children with CP with a positive attitude, so as to promote the rehabilitation of children with CP and enable children with CP to return to family, school and society.

## Author contributions

**Conceptualization:** Ling Wan, Qiong He.

**Data curation:** Lin Jiang, Minghui Yin.

**Formal analysis:** Ling Wan, Sha Chen.

**Methodology:** Ling Wan, Qiong He.

**Software:** Ling Wan.

**Writing – original draft:** Ling Wan.
